# Predictable Roles of Peripheral IgM Memory B Cells for the Responses to Anti-PD-1 Monotherapy Against Advanced Non-Small Cell Lung Cancer

**DOI:** 10.3389/fimmu.2021.759217

**Published:** 2021-11-24

**Authors:** Liliang Xia, Limin Guo, Jin Kang, Yi Yang, Yaxian Yao, Weimin Xia, Ruiming Sun, Shun Zhang, Wenfeng Li, Yuer Gao, Hongyan Chen, Ziming Li, Jinji Yang, Shun Lu, Ying Wang

**Affiliations:** ^1^ Department of Shanghai Lung Cancer Center, Shanghai Chest Hospital, Shanghai Jiao Tong University, Shanghai, China; ^2^ Department of Immunology and Microbiology, Shanghai Institute of Immunology, Shanghai Jiao Tong University School of Medicine, Shanghai, China; ^3^ Department of Genetic Engineering, School of Life Sciences and Institute of Biomedical Sciences, Fudan University, Shanghai, China; ^4^ Guangdong Lung Cancer Institute, Guangdong Provincial Key Laboratory of Translational Medicine in Lung Cancer, Guangdong Provincial People’s Hospital, Guangdong Academy of Medical Sciences, School of Medicine, Guangzhou, China

**Keywords:** immune checkpoint inhibitors, PD-1, peripheral IgM^+^ memory B cells, response prediction, advanced NSCLC

## Abstract

Tumor-infiltrating B cells and tertiary lymphoid structures have been identified to predict the responses to immune checkpoint inhibitors (ICIs) in cancer immunotherapy. Considering the feasibility of sample collection, whether peripheral B cell signatures are associated with the responses to ICI therapy remains unclear. Herein, we have defined peripheral B cell signatures in advanced non-small cell lung cancer (NSCLC) patients receiving anti-PD-1 monotherapy and investigated their associations with clinical efficacy. It was found that the percentages of B cells before the treatment (baseline) were significantly higher (*P* = 0.004) in responder (R, n = 17) than those in non-responder (NonR, n = 33) NSCLC patients in a discovery cohort. Moreover, the percentages of baseline IgM^+^ memory B cells were higher (*P* < 0.001) in R group than those in NonR group, and associated with a longer progression free survival (PFS) (*P* = 0.003). By logistic regression analysis peripheral baseline IgM^+^ memory B cells were identified as an independent prognostic factor (*P* = 0.002) for the prediction of the responses to anti-PD-1 monotherapy with the AUC value of 0.791, which was further validated in another anti-PD-1 monotherapy cohort (*P* = 0.011, n = 70) whereas no significance was observed in patients receiving anti-PD-L1 monotherapy (*P* = 0.135, n = 30). Therefore, our data suggest the roles of peripheral IgM^+^ memory B cells in predicting the responses to anti-PD-1 treatment in Chinese advanced NSCLC patients.

## Introduction

Immune checkpoint inhibitors (ICIs) targeting programmed cell death1 (PD­1) and its ligand (PD-L1) have greatly improved therapeutic outcomes in multiple carcinomas including advanced non-small cell lung cancer (NSCLC). However, the limited benefit population of ICI monotherapy makes it necessay to screen predictive biomarkers for stratifying the patients. Currently, PD-L1 expression on tumor cells has been used in clinic as an indicative biomarker (1). In addition, a high tumor mutation burden (2, 3), intratumor immunological signatures (4, 5), as well as increased diversity of gut microbiota (6, 7) are also reported to be associated with better responses to ICI treatment. Nevertheless, due to the difficulties in the collection of tumor biopsies from cancer patients and insufficient biomarkers to identify benefit patients, it is still necessary to investigate novel indicators associated with the responses to ICI treatment.

Peripheral blood is the most widely used specimen in clinical diagnosis. ICI treatment is demonstrated to affect the peripheral immune profiles. For instance, PD-1 blockade induces Ki67 expression in a subset of peripheral PD-1^+^CD8^+^ T cells and the expansion of neoantigen-specific T cell clones after anti-PD-1 treatment, which suggests the restoration of systematic anti-tumor immunity of CD8^+^ T cells after receiving ICI immunotherapy (8, 9). Therefore, several peripheral CD8^+^ T cell signatures have been reported to be associated with clinical outcomes of anti-PD-1/PD-L1 immunotherapy (8, 10). Our previous study also illustrated that high percentages of IFN-γ-producing naïve CD4^+^ T cells and PD-1^+^CD4^+^ memory T cells were associated with better response to anti-PD-1 treatment in NSCLC patients (11). Recently, B cells and tertiary lymphoid structures (TLSs) in the tumor microenvironment (TME) are identified to promote the responses to ICI treatment, illustrating the significance of B cells in anti-PD-1/PD-L1 immunotherapy (12–14). Whether systematic B cell signatures are associated with the responses to ICI therapy needs to be further explored.

In this study, peripheral B cell signatures from advanced NSCLC patients receiving anti-PD-1/PD-L1 monotherapy were systematically assessed by multiplex flow cytometry. Peripheral IgM^+^ memory B cells were identified to be associated with the responses to anti-PD-1 monotherapy in advanced NSCLC patients, providing the evidence on peripheral B cell signatures as a potential biomarker for stratifying the patients before the treatment.

## Methods

### Patients

A total of 150 advanced NSCLC patients receiving ICI monotherapy were recruited in this study from Shanghai Chest Hospital affiliated to Shanghai Jiaotong University (n = 115) and Guangdong Provincial People’s Hospital (n = 35) from September 2017 to October 2020. Among them, 50 patients receiving nivolumab monotherapy after the failure of one to two prior systemic chemotherapies in Shanghai Chest Hospital were designated as a discovery cohort (cohort 1, **Table 1**). The advanced NSCLC patients received 240 mg nivolumab every two weeks. The responses to nivolumab treatment were evaluated every 8 weeks according to the RECIST 1.1 criteria. Accordingly, those whose tumor growth increased at least 20% were defined as progression diseases (PD), whereas decreased up to 30% were defined as partial response (PR) or stable disease (SD). Patients with PD within 180 days were annotated as non-responders (NonR) and those with SD + PR within 180 days were as responders (R). In addition, 70 advanced NSCLC patients receiving anti-PD-1 monotherapy (cohort 2, **Table 1**) and 30 advanced NSCLC patients receiving anti-PD-L1 monotherapy (cohort 3, **Table 1**) were recruited as independent validation cohorts, respectively. This study was approved by the Ethics Committee of Shanghai Chest Hospital (Number KS1732). All patients were informed of the study and consented to the enrollment. All the procedures were conducted in accordance with the Declaration of Helsinki.

**Table 1:** Clinical manifestations of advanced NSCLC patients receiving ICI treatments.

Characteristics	Cohort 1 anti-PD-1 monotherapy (n = 50)	Cohort 2 anti-PD-1 monotherapy (n = 70)	Cohort 3 anti-PD-L1 monotherapy (n = 30)
Age, year			
Median	61	61	62
Sex, n (%)			
Male	41 (82.0)	57 (81.4)	26 (86.7)
Female	9 (18.0)	13 (18.6)	4 (13.3)
History, n (%)			
Squamous	17 (34.0)	23 (32.9)	12 (40.0)
Non-squamous	33 (66.0)	47 (67.1)	18 (60.0)
Smoking status, n (%)			
Smoker	39 (78.0)	44 (62.9)	23 (76.7)
Nonsmoker	11 (22.0)	20 (28.6)	7 (23.3)
Unknown	0 (0.0)	6 (8.5)	0 (0.0)
Disease stage, n (%)			
III	8 (16.0)	12 (17.1)	1 (3.3)
IV	42 (84.0)	58 (82.9)	29 (96.7)
EGFR mutation, n (%)			
Yes	6 (12.0)	7 (10.0)	2 (6.7)
No	37 (74.0)	44 (62.9)	28 (93.3)
Unknown	7 (14.0)	19 (27.1)	0 (0.0)
Treatment, n (%)			
First-line	0 (0.0)	19 (27.1)	15 (50.0)
Second-line	45 (90.0)	33 (47.1)	10 (33.3)
More than second	5 (10.0)	18 (25.8)	5 (16.7)
Response			
R	17 (34.0)	24 (34.3)	15 (50.0)
NonR	33 (66.0)	46 (65.7)	15 (50.0)
Median PFS (95% CI, days)	70 (21-119)	83 (44-122)	170 (60-280)

### Multi-Color Flow Cytometry

Whole blood was collected with anticoagulation and peripheral blood mononuclear cells (PBMCs) were isolated by density gradient centrifugation using Lymphoprep™ reagent (Axis-shield, Oslo, Norway) according to the manufacturer’s instructions. Cells were stained with fluorochrome-conjugated monoclonal antibodies (mAbs) for multiplex flow cytometry analysis. Briefly, 1×10^6^ PBMCs were resuspended in 100 μL FACS buffer (phosphate buffer saline supplemented with 2% fetal bovine serum) (Millipore, Bedford, MA, USA) and incubated with the mixtures of fluorochrome-conjugated mAbs targeting multiple cell surface markers including human CD3, CD8, CD19, CD16, CD27, CD38, CD24, PD-1, PD-L1, IgG and IgM (listed in **Table S1**). After incubation for 40 min at 4°C, cells were washed twice with 1 mL FACS buffer and resuspended in 200 μL FACS buffer. Cells were immediately acquired on LSR Fortessa (BD Pharmingen, San Diego, CA) and data analysis was performed with FlowJo software version 10 (Tree Star Inc., Ashland, Oregon).

### Statistical Analyses

All the data were represented by mean ± standard error of mean (S.E.M). Statistical analyses were conducted by using SPSS 19.0 software (IBM SPSS Software, Armonk, NY, USA) or GraphPad Prism 6.0 (GraphPad Software Inc., San Diego, CA, USA). Tests for the differences between two groups were performed using a Wilcoxon test. Survival curves were performed by using the Kaplan-Meier method. The median frequencies were chosen as the cutoff to define the high and low group. *P*-values were calculated by the log-rank statistics in the Kaplan-Meier analyses. Multivariate analyses of the signatures associated with the responses to anti-PD-1 therapy were performed by using the logistic regression. The receiver operating characteristic (ROC) curves were constructed by plotting the true positive rate (sensitivity) against the false positive rate (1-specificity). *P* values were two-sided and *P* < 0.05 was considered statistically significant.

## Results

### Patient Characteristics

A total of 120 advanced NSCLC patients receiving anti-PD-1 monotherapy and 30 patients receiving anti-PD-L1 monotherapy were recruited in this study (**Table 1**). Among those receiving anti-PD-1 monotherapy, 50 patients with nivolumab monotherapy were designated as a discovery cohort (cohort 1, **Table 1**) and another 70 patients were as a validation cohort (cohort 2, **Table 1**). Median days of the progression free survival (PFS) in cohort 1 and cohort 2 were 70 days (95% CI: 21-119 days) and 83 days (95% CI: 44-122 days), respectively according to the RECIST 1.1 criteria with no significant difference. In cohort 1, 17 patients (34.0%) with no disease progression in more than 180 days after nivolumab monotherapy were classified as R group, whereas 33 patients (66.0%) who had disease progression within 180 days were defined as NonR group. There were no significant differences in age, gender, smoking status and tumor stages *etc.* among R and NonR patients in cohort 1 (**Table S2**). Additionally, thirty patients receiving anti-PD-L1 monotherapy (cohort 3, **Table 1**) were included as another validation cohort.

### High Percentages of Peripheral CD19^+^ B Cells at the Baseline in NSCLC Patients Are Associated With Good Responses to Anti-PD-1 Monotherapy

**Figure 1:**
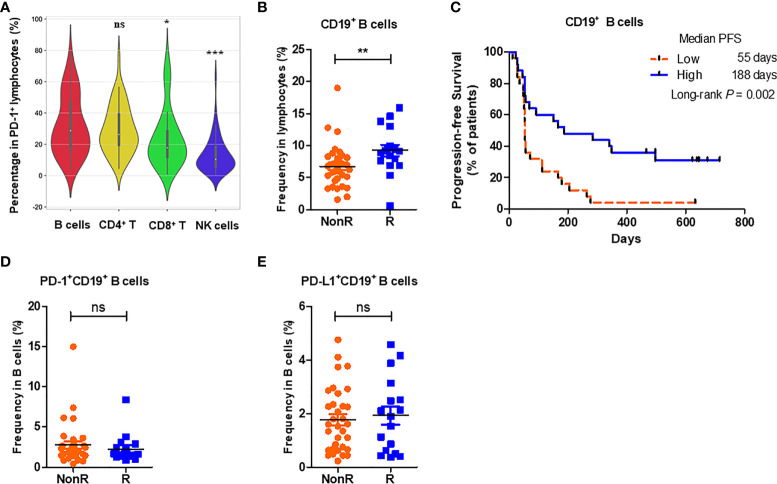
High percentages of CD19^+^ B cells at the baseline are associated with better responses to nivolumab monotherapy. **(A)** The average percentages of B cells (CD3^-^CD19^+^), CD4^+^ T cells (CD3^+^CD8^-^), CD8^+^ T cells (CD3^+^CD8^+^) and NK cells (CD3^-^CD16^+^) in PD-1^+^ lymphocytes in the periphery of advanced NSCLC patients (n = 50) before nivolumab treatment. **(B)** Comparison of the frequencies of CD19^+^ B cells between R (n = 17) and NonR (n = 33) NSCLC patients receiving anti-PD-1 monotherapy at the baseline. **(C)** Kaplan-Meier analysis of the associations of the percentages of CD19^+^ B cells with the PFS values. **(D, E)** Comparison of the frequencies of PD-1^+^CD19^+^ B cells **(D)** and PD-L1^+^CD19^+^ B cells **(E)** between R (n = 17) and NonR (n = 33) NSCLC patients at the baseline. The Wilcoxon test was used to analyze the differences between two groups. Survival curves were plotted by using the Kaplan-Meier method with the median as the cutoff to define the high and low group. *P*-values were calculated by the log-rank statistics in Kaplan-Meier analyses. ***P < 0.001, **P < 0.01, *P < 0.05, ns, *P* > 0.05.

Previous investigates have addressed B cells and TLSs in the TME to promote the response to ICI immunotherapy (12–14). Considering the feasibility of peripheral blood in sample collection, we therefore investigated B cell signatures in advanced NSCLC patients and their associations with the responses to nivolumab monotherapy. Firstly, the expression profiles of PD-1 positive lymphocytes in the periphery of 50 advanced NSCLC patients were measured by multiplex flow cytometry. It was showed that among PD-1^+^ lymphocytes the percentages of B cells (31.90% ± 2.69%) were comparable with those of CD4^+^ T cells (29.35% ± 2.15%, *P* = 0.596), but significantly higher than those of CD8^+^ T cells (23.73% ± 2.61%, *P* = 0.011) and NK cells (13.35% ± 1.62%, *P* < 0.001) (**Figure 1A**). Peripheral B cells therefore accounted for a main proportion in PD-1 expressing lymphocytes. Subsequently, we compared the percentages of peripheral B cells at the baseline between R and NonR NSCLC patients in cohort 1. It was found that the percentages of CD19^+^ B cells in peripheral lymphocytes were higher in R (n = 17) than those in NonR patients (n = 33) (*P* = 0.004) (**Figure 1B**). More significantly, patients with high percentages of peripheral CD19^+^ B cells (median percentage as a cutoff value) showed a significantly longer PFS (median PFS: high *vs.* low = 188 *vs.* 55 days, *P* = 0.002) (**Figure 1C**). However, the percentages of PD-1 (**Figure 1D**) and PD-L1 expressing CD19^+^ B cells among periphery B cells (**Figure 1E**) were comparable between R and NonR patients, respectively. These results indicate that high percentages of peripheral CD19^+^ B cells are associated with better responses to nivolumab monotherapy in advanced NSCLC patients.

### The Profiles of Peripheral IgM^+^ B Cell Subsets In Advanced NSCLC Patients Receiving Nivolumab Monotherapy

**Figure 2:**
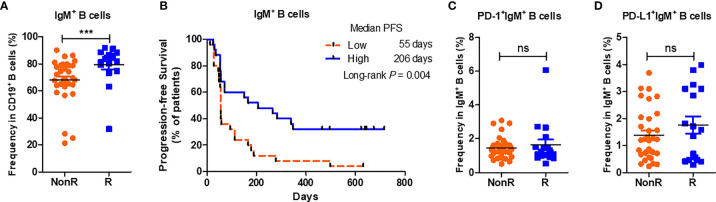
High percentages of IgM positive cells in B cells at the baseline facilitate the response to anti-PD-1 treatment. **(A)** Comparison of the percentages of IgM^+^ B cells between R (n = 17) and NonR (n = 33) NSCLC patients receiving anti-PD-1 monotherapy at the baseline. **(B)** Kaplan-Meier analysis of the associations of the percentages of IgM^+^ B cells with the PFS values. **(C, D)** Comparison of the frequencies of PD-1^+^IgM^+^ B cells **(C)** and PD-L1^+^IgM^+^ B cells **(D)** between R (n = 17) and NonR (n = 33) NSCLC patients at the baseline. The Wilcoxon test was used to analyze the differences between two groups. Survival curves were plotted by using the Kaplan-Meier method with the median as the cutoff to define the high and low group. *P*-values were calculated by the log-rank statistics in Kaplan-Meier analyses. ***P < 0.001, ns, *P* > 0.05.

Peripheral B cells can be subgrouped into certain subsets with diverse functional implementations (15, 16). To further investigate whether B cell subsets are associated with the responses to nivolumab monotherapy, we firstly compared the percentages of IgM^+^ and IgG^+^ B cells, two subsets with different functional definition (17), between R and NonR patients. Notably, the percentages of IgM^+^ B cells were higher in R patients than those in NonR patients (*P* < 0.001, **Figure 2A**). High percentages of IgM^+^ B cells were associated with a long PFS (median PFS: high *vs.* low = 206 *vs.* 55 days, *P* = 0.004) (**Figure 2B**). However, no significant differences in PD-1 and PD-L1 expressions on IgM^+^ B cells were observed between R and NonR patients either (**Figures 2C, D**). On the contrary, the percentages of IgG expressing B cells were lower in R patients (*P* = 0.032, **Figure S1B**) but with no associations to a better outcome (*P* = 0.312, **Figure S1C**).

**Figure 3:**
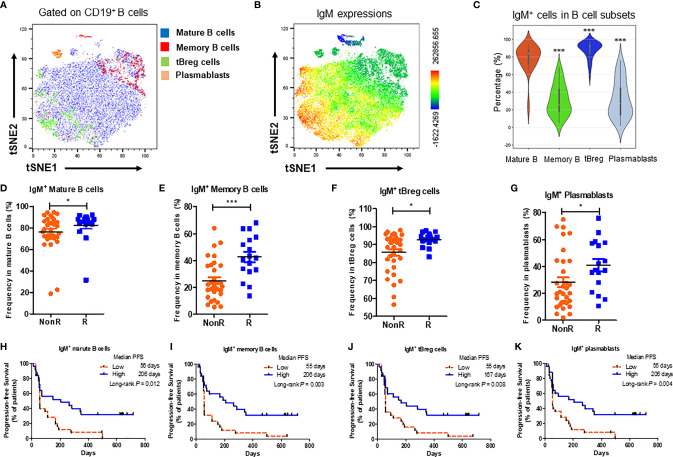
High expressions of IgM on B cells subsets at the baseline are related to better responses to anti-PD-1 treatment. **(A)** Exemplified t-distributed stochastic neighbor embedding (t-SNE) visualization of B cell subsets including mature B (CD24^+^CD38^-^CD19^+^ B), memory B (CD27^+^CD19^+^ B), tBreg (CD24^++^CD38^+^CD19^+^ B) and plasmablasts (CD24^-^CD38^+^CD19^+^ B) from 13 NSCLC patients. **(B)** IgM expressions on B cell subsets by t-SNE analysis. **(C)** Comparison of IgM expressions between mature B cells, memory B cells, tBreg cells and plasmablasts in NSCLC patients receiving anti-PD-1 therapy at the baseline (n = 50). **(D–G)** Comparison of the percentages of IgM^+^ mature B cells **(D)**, IgM^+^ memory B cells **(E)**, IgM^+^ tBreg cells **(F)** and IgM^+^ plasmablasts cells **(G)** between R (n = 17) and NonR (n = 33) NSCLC patients receiving anti-PD-1 monotherapy. **(H–K)** Kaplan-Meier analysis of associations of the percentages of IgM^+^ mature B cells **(H)**, IgM^+^ memory B cells **(I)**, IgM^+^ tBreg cells **(J)** and IgM^+^ plasmablasts cells **(K)** with the PFS values. The Wilcoxon test was used to analyze the differences between two groups. Survival curves were plotted by using the Kaplan-Meier method with the median as the cutoff value to define the high and low group. *P*-values were calculated by the log-rank statistics in Kaplan-Meier analyses. ***P < 0.001, *P < 0.05.

B cells could be subgrouped into mature B cells (CD24^+^CD38^-^CD19^+^), memory B cells (CD27^+^CD19^+^), transitional regulatory B cells (CD24^++^CD38^+^CD19^+^) (tBreg) and plasmablasts (CD24^-^CD38^+^CD19^+^) (15, 16) (**Figure S2**). Differential expressions of IgM on B cell subsets were plotted in the t-distributed stochastic neighbor embedding (t-SNE) dimensionality reduction analysis in 13 advanced NSCLC patients of cohort 1 (**Figures 3A, B**). It was found that IgM expressions were high in mature B cells and tBreg cells, but low in memory B cells and plasmablasts (**Figure 3C**). It was notable that the percentages of IgM expressing mature B cells (**Figure 3D**), memory B cells (**Figure 3E**), tBreg cells (**Figure 3F**) and plasmablasts (**Figure 3G**) were higher in R patients than those in NonR patients, respectively. In line with the results from IgM^+^ B cells, high IgM expressions on B cell subsets were associated with a long PFS in the cohort 1 (**Figures 3H–K**). Collectively, our results further suggest that patients with more percentages of IgM expressing B cells as well as differentiation subsets at the baseline are inclined to respond better to nivolumab immunotherapy.

### Baseline IgM^+^ Memory B Cells Is Predictable for the Responses to Nivolumab Monotherapy

**Figure 4:**
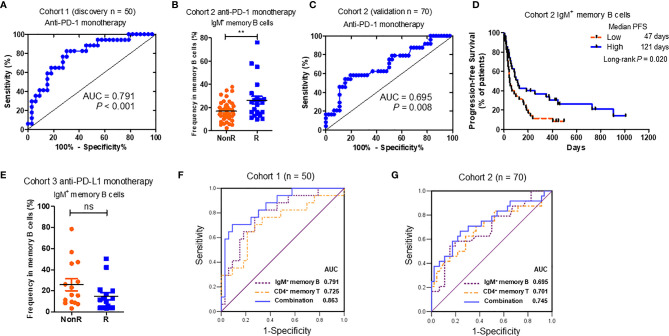
Significance of baseline IgM^+^ memory B cells in predicting the response to anti-PD-1 monotherapy. **(A)** The Receiver Operating Characteristic (ROC) analyses of IgM^+^ memory B cell percentages in discovery cohort (cohort 1 in **Table 1**, n = 50). **(B)** Comparison of the percentages of IgM^+^ memory B cells between R (n = 24) and NonR (n = 46) advanced NSCLC patients with anti-PD-1 monotherapy in an independent validation cohort (cohort 2 in **Table 1**, n = 70). **(C)** The ROC analysis of IgM^+^ memory B cell percentages in validation cohort (cohort 2 in **Table 1**, n = 70). **(D)** The Kaplan-Meier analysis of the associations of the percentages of IgM^+^ memory B cells with the PFS in cohort 2. **(E)** Comparison of the percentages of IgM^+^ memory B cells between R (n = 15) and NonR (n = 15) advanced NSCLC patients with anti-PD-L1 monotherapy in an independent validation cohort (cohort 3 in **Table 1**, n = 30). **(F, G)** The ROC analyses of baseline CD19^+^ memory B combined with CD4^+^ memory T cell percentages in discovery cohort 1 **(F)** and validation cohort 2 **(G)**. The Wilcoxon test was used to analyze the differences between two groups. Survival curves were plotted by using the Kaplan-Meier method using median as the cutoff to define the high and low group. *P*-values were calculated by the log-rank statistics in Kaplan-Meier analyses. **P < 0.01, ns, *P* > 0.05.

Aforementioned results revealed that certain peripheral B cell signatures at the baseline were associated with good responses to nivolumab treatment in Chinese advanced NSCLC patients. We further used multivariate logistic regression to evaluate their predictive values. Among all the B cell signatures associated with longer PFS values (**Table 2**), the percentages of IgM^+^ memory B cells were the most significant in cohort 1 (*P* = 0.002) (**Table 2**). According to the ROC curve analysis, the Area Under Curve (AUC) values of IgM^+^ memory B cells reached 0.791 in cohort 1 (**Figure 4A**). What is more, the predictive significance of IgM^+^ memory B cells was validated in another cohort of advanced NSCLC patients receiving anti-PD-1 monotherapy (cohort 2 in **Table 1**, n = 70). In the validation cohort, higher percentages of baseline IgM^+^ memory B cells were detected in R group than those in NonR group (*P* = 0.011) (**Figure 4B**). The AUC value reached 0.695 (**Figure 4C**). NSCLC patients with high percentages of IgM^+^ memory B cells displayed a longer PFS (median PFS: high *vs.* low = 121 *vs.*47 days) (*P* = 0.020) (**Figure 4D**) as well. Significantly, a combination of baseline IgM^+^ memory B cell and CD4^+^ T memory cell percentages obtained from our previous study (11) (**Table S3**) achieved higher sensitivity for response prediction to anti-PD-1 treatment both in discovery cohort (AUC = 0.863) (**Figure 4F**) and validation cohort (AUC = 0.745) (**Figure 4G**) when compared to individual signature. However, the percentages of IgM^+^ memory B cells were comparable between R and NonR NSCLC patients receiving anti-PD-L1 monotherapy (cohort 3 in **Table 1**, n = 30) (**Figure 4E**), which suggests the distinct role of IgM^+^ memory B cells in predicting the efficacy of anti-PD-1 and anti-PD-L1 therapies. Our results thus indicate that the percentage of peripheral IgM^+^ memory B cells at the baseline is a novel indicator for predicting the responses to anti-PD-1 monotherapy in advanced NSCLC patients.

**Table 2:** Univariate and multivariate logistic regression analyses of B cell signatures in the prediction of the responses to anti-PD-1 monotherapy.

Factors	Univariate (Kaplan–Meier test)		Multivariate (Logistic regression)	
	*P* value	HR (95% CI)	*P* value	HR (95% CI)
CD19^+^ B cells	0.002	2.97 (1.52-5.84)	0.188	
IgM^+^ B cells	0.004	2.63 (1.36-5.10)	0.378	
IgM^+^ Mature B cells	0.012	2.33 (1.21-4.50)	0.633	
IgM^+^ Memory B cells	0.003	2.72 (1.39-5.30)	0.002	1.07 (1.03-1.12)
IgM^+^ tBreg cells	0.004	2.68 (1.38-5.20)	0.073	
IgM^+^ Plasmablasts	0.008	2.44 (1.27-4.70)	0.667	

### Dynamics of PD-1 and PD-L1 Expressions on IgM^+^ Memory B Cells During Nivolumab Treatment

**Figure 5:**
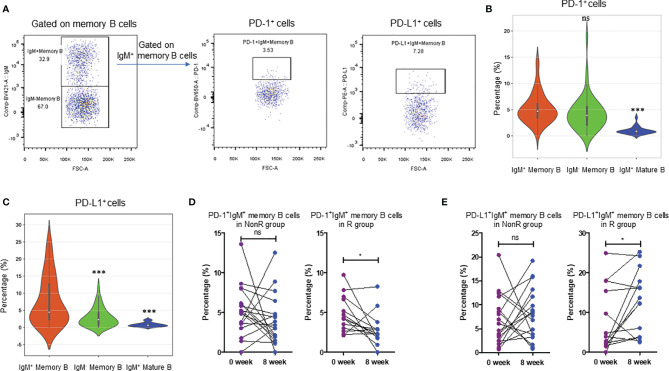
Dynamics of peripheral PD-1 and PD-L1-expressing IgM^+^ memory B cells in R and NonR patients receiving anti-PD-1monotherapy. **(A)** Gating strategy for PD-1^+^ and PD-L1^+^ cells in IgM^+^ memory B cells by flow cytometry. **(B)** Comparisons of PD-1 expressions on IgM^+^ memory B cells, IgM^-^ memory B cells and IgM^+^ mature cells. **(C)** Comparisons of PD-L1 expressions on IgM^+^ memory B cells, IgM^-^ memory B cells and IgM^+^ mature cells. **(D, E)** Dynamics of the percentages of PD-1^+^IgM^+^ memory B cells **(D)** and PD-L1^+^IgM^+^ memory B cells **(E)** at the baseline and 8 weeks after nivolumab treatment in R (n = 14) and NonR (n = 18) patients. The paired-*Student t* test was used to analyze the differences between two groups as well as in baseline and after the treatment. ***P < 0.001, *P < 0.05, ns, *P* > 0.05.

Since baseline IgM^+^ memory B cells exhibited the potential in predicting anti-PD-1 treatment, we further investigated the expression profiles of PD-1 and PD-L1 on IgM^+^ memory B cells (**Figure 5A**). Our data showed that the percentages of PD-1 positive cells in IgM^+^ memory B cells were comparable to those in IgM^-^ memory B cells (*P* = 0.803), but significantly higher than those in IgM^+^ mature B cells (*P* < 0.001) (**Figure 5B**). In addition, the percentages of PD-L1 expressing cells on IgM^+^ memory B cells were significantly higher than those in IgM^-^ memory B cells (*P* < 0.001) and IgM^+^ mature B cells (*P* < 0.001) (**Figure 5C**), respectively.

We also analyzed the dynamics of IgM^+^ memory B cell percentages at the baseline and 8 weeks after anti-PD-1 treatment. There were no significant changes of IgM^+^ memory B cell percentages from the baseline to 8 weeks after the treatment in R and NonR patients (**Figures S3G, H**). However, the percentages of PD-1^+^IgM^+^ memory B cells were reduced in R patients (*P* = 0.028) at 8 weeks whereas no statistical change was observed in NonR patients (*P* = 0.310) (**Figure 5D**). On the contrary, the percentages of PD-L1^+^IgM^+^ memory B cells were increased in R patients (*P* = 0.030) at 8 weeks but stable in NonR patients (*P* = 0.476) (**Figure 5E**). Considering the high expression of PD-1 on IgM^+^ memory B cells, the reduction of PD-1^+^IgM^+^ memory B cells after 8 weeks in R patients might represent efficient interaction of nivolumab antibodies with unique B cell subset during the treatment.

## Discussion

T cells including CD8^+^ and CD4^+^ T cells play significant roles in mediating durable anti-tumor immunity (18–21), making them feasible in predicting the responses to ICI immunotherapy in clinic (8, 11, 22–24). Although B cells are mostly dedicated to humoral immunity against microbial infections, recent findings also illustrate response prediction significance of tumor-infiltrating B cells and TLSs in the patients with melanoma and sarcoma upon ICI treatment (12–14). In the present study, we have investigated the potential of peripheral B cells in predicting the responses to anti-PD-1/PD-L1 treatment in Chinese advanced NSCLC patients. In line with the findings in the TME, our results show that the percentage of CD19^+^ B cells at the baseline is higher in R than that in NonR group. What is more, the percentage of IgM^+^ memory B cells at the baseline is the most potential signature in predicting the responses to anti-PD-1 monotherapy.

B cells exert anti-tumor immunity mainly through antigen presentation and cytokine secretion for T cell activation (17, 25, 26). Although the exact mechanisms of B cells in facilitating the responses to ICI therapy need to be further investigated, it has been proposed that B cells as well as TLSs in tumor regions played critical roles in the activation of regional tumor-specific T cells through presenting tumor antigens (12). We also compared the infiltrating B cells in tumor biopsies between R (n = 3) and NonR (n = 12) patients before the treatment. No significant difference was observed between these two groups (**Figure S4**), which is similar to the results by Helmink BA et al. (13). Other studies also revealed the properties of infiltrating B cells associated with the responses to ICI treatment. For instance, clonal counts for both immunoglobulin heavy and light chains as well as BCR diversity were increased in the responders than those in the non-responders of melanoma patients upon neoadjuvant ICI therapy (13). Additionally, tumor associated B cells were found to sustain the inflammation in melanoma, which also facilitated the responses to ICI therapy (27). These results therefore provide the possible mechanism of infiltrating B cells on anti-tumor immunity in the TME.

In this study, we have demonstrated that high percentages of peripheral CD19^+^ B cells before the treatment were associated with a longer PFS in advanced NSCLC patients receiving nivolumab monotherapy. Compared to CD8^+^ T cells or NK cells, B cells account for a large proportion in PD-1 expressing lymphocytes (**Figure 1A**). PD-1 is previously reported to be engaged in regulating B cell-mediated humoral immunity (28, 29). We have also reported that blockade of PD-1 in mice augmented humoral immunity with the accumulation of germinal center B cells (GCBs) and memory B cells in the spleens together with elevated percentages of plasma cells (30). We therefore determined IgG and IgM levels in the patients receiving anti-PD-1 treatment in some patients of cohort 1, where we deduced that IgG and/or IgM might be also related to the efficacy of ICIs treatment. However, both IgG and IgM before anti-PD-1 monotherapy were comparable between R and NonR groups (**Figure S5**). Interestingly, IgM^+^ B cells highly express HLA-DR when compared to other B cell subsets (**Figure S6B**). These data suggests that the effects of PD-1 blockade on human B cells might be more related to their antigen presentation capacity with the upregulation of MHC molecules (12) rather than their differentiation into antibody-producing plasma cells.

What is more, logistical regression analysis screened out IgM^+^ memory B cells as a noval indicator to predict the efficacy of anti-PD-1 monotherapy. Memory B cells are a B cell subset which is responsible for the maintenance of memory response upon microbial infection. Based on IgG and IgM expressions, they can be subdivided into long-lived IgM^+^ and class-switched IgG^+^ memory B cells. IgM^+^ memory B cells are demonstrated to play important roles in lasting long term immunity with less turnover (31). It has been reported that when encountering the same or similar antigens IgM^+^ memory B cells may reenter GCs to undergo further affinity maturation and isotype switch (32, 33). This subpopulation in the circulation has been associated with the outcome of the infections such as COVID-19 (34, 35). We have found that they expressed high level of PD-1 when compared to IgM^+^ mature B cells (**Figure 5B**). In addition, PD-L1 expression was much higher on IgM^+^ mature B cells than on both IgM^-^ (mainly IgG^+^) memory B cells and IgM^+^ mature B cells (**Figure 5C**). More interestingly, upon anti-PD-1 treatment, there exhibited the decrease in peripheral PD-1^+^IgM^+^ memory B cells in R group than in NonR group whereas no similar trend was observed in PD-1^+^IgM^-^ memory B cells (data not shown). This might be explained that PD-1^+^IgM^+^ memory B cells might be one of the targets of anti-PD-1 antibody, which is similar to PD-1^+^CD8^+^ T cells reported previously (8, 10). Herein, we did not define how IgM^+^ memory B cells modulate anti-PD-1 regimen-mediated anti-tumor immunity in NSCLC patients. Given that IgM^+^ memory B cells are the main subpopulation in the secondary responses with high expressions of HLA-DR (**Figure S6C**), they might be engaged in promoting anti-tumor immunity through remodeling the capacity of antigen presentation upon nivolumab immunotherapy. In the future, whether IgM^+^ memory B cells migrate into the TME and how they interplay with T cells to enhance anti-tumor immunity merit further investigation. This might shed light on the mechanisms of B cells contributing to enhance ICI therapy.

In this study, since all the patients recruited have received at least one or two-rounds of chemotherapy before anti-PD-1 monotherapy, we also analyzed the effects of prior chemotherapy on peripheral B cells. It was showed that the prior chemotherapy has little effects on the proportions of peripheral CD19^+^ B cells at the beginning of anti-PD-1 monotherapy as well as the prognosis (**Figure S7**). Moreover, there was no significant difference in peripheral CD19^+^ B cell percentages between R and NonR patients at 2 and 8 weeks after the treatment (**Figures S3A, C**). No obvious changes were observed in the percentages of peripheral CD19^+^ B cells from the baseline to 8 weeks after immunotherapy either (**Figures S3E, F**). However, both peripheral CD19^+^ B cells and IgM^+^ memory B cell at the baseline were not associated with a longer OS (**Figure S8**). This might be due to the fact that B cell subsets are not the populations undergoing direct cytotoxicity against tumor cells. They are more likely to perform indirect function such as presenting tumor antigen for T cell activation, which might be more related to the PFS rather than OS.

Another interesting observation we got from this study is that the prediction roles of peripheral IgM^+^ memory B cells for anti-PD-1 monotherapy is not suitable for anti-PD-L1 monotherapy (**Figure 4E**). One of the reasons might be the sample size in anti-PD-L1 monotherapy cohort (n = 30). Another reason might be due to the different mechanisms of anti-PD-1 and anti-PD-L1 treatment against tumor. This is also demonstrated by our previous studies both in CD4^+^ T cell subsets (11) or metabolic biomarker signatures (36) for response prediction of ICI treatment in the same cohort. Our findings warrant further exploration in the prospective study with a larger advanced NSCLC population, as well as other types of tumors (37).

In summary, our study identified peripheral IgM^+^ memory B cells with high PD-1 and PD-L1 expressions as an alternative potential indicator to predict the responses to anti-PD-1 monotherapy in Chinese advanced NSCLC. Since plenty of the signatures in the periphery have been identified independently including CD8^+^ T cells (8, 10), CD4^+^ T cells (24, 38), neutrophil-to-lymphocyte ratio (NLR) (39), monocytes (40), IL-8 (41, 42), LDH (43), as well as ctDNA (43, 44). How to integrate different signatures to establish multiparametric approaches to improve prediction efficacy will be significant in the future.

## Data Availability Statement

The raw data supporting the conclusions of this article will be made available by the authors, without undue reservation.

## Ethics Statement

All samples were collected in accordance with the Ethics Committee of Shanghai Chest Hospital-approved protocol (Number KS1732). All patients have provided written consent prior to blood collection.

## Author Contributions

YW, JY, and SL conceived and designed the study. LX, LG, WX, RS, and SZ performed the flow cytometric experiments. JK, YY, YXY, WL, and YG were working on the clinical sample collection. LX, LG, JK, and YW performed data analysis. HC and ZL offered assistance and helpful discussions for data analysis. YW, SL, JY, and LX interpreted the results and drafted the manuscript. All authors contributed to the article and approved the submitted version.

## Funding

This research was funded by the National Key R&D Program of China (2016YFC1303300), National Natural Science Foundation of China (82073152, 81802264, 82030045), Technology Innovation Program of Shanghai (19411950500), Talent Training Program of Shanghai Chest Hospital in 2019, Incubation Project Plan for Research in Shanghai Chest Hospital (2019YNJCM07), and Shanghai Chest Hospital Project of Collaborative Innovative Grant (YJT20191015).

## Conflict of Interest

The authors declare that the research was conducted in the absence of any commercial or financial relationships that could be construed as a potential conflict of interest.

## Publisher’s Note

All claims expressed in this article are solely those of the authors and do not necessarily represent those of their affiliated organizations, or those of the publisher, the editors and the reviewers. Any product that may be evaluated in this article, or claim that may be made by its manufacturer, is not guaranteed or endorsed by the publisher.
