# Supplementary material for: Predictable Roles of Peripheral IgM Memory B Cells for the Responses to Anti-PD-1 Monotherapy Against Advanced Non-Small Cell Lung Cancer
Source: Front Immunol. 2021 Nov 24;12:759217. doi: 10.3389/fimmu.2021.759217 (PMC8652218; doi:10.3389/fimmu.2021.759217)
Supplement: Supplementary file 1 [file DataSheet_1.docx]

**Predictable roles of peripheral IgM^+^ memory B cells for the responses to anti-PD-1 monotherapy against advanced non-small cell lung cancer**

Liliang Xia^1,2†^, Limin Guo^3†^, Jin Kang^4†^, Yi Yang^1^, Yaxian Yao^1^, Weimin Xia^2^, Ruiming Sun^2^, Shun Zhang^2^, Wenfeng Li^4^, Yuer Gao^4^, Hongyan Chen^3^, Ziming Li^1^, Jinji Yang^4*^, Shun Lu^1*^, Ying Wang^2*^

**Supplementary materials:**


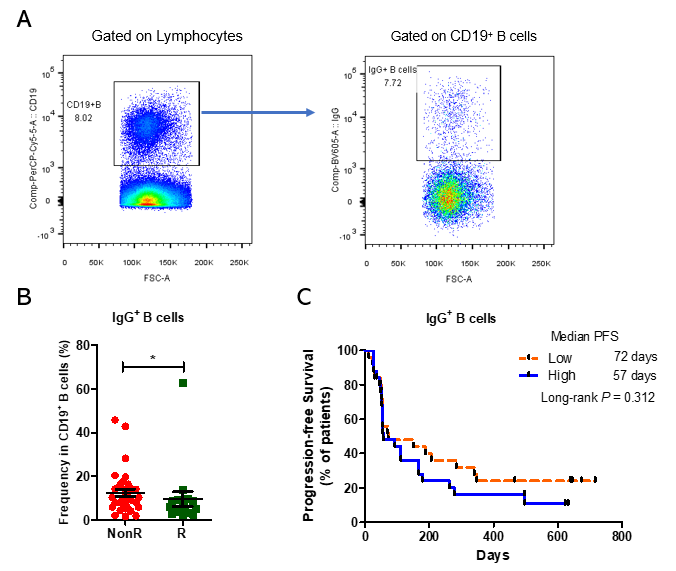


**FIGURE S1** Comparison of the percentages of IgG^+^ B cells between R and NonR NSCLC patients receiving anti-PD-1 monotherapy at the baseline. **(A)** The gating strategy of IgG^+^ B cells in CD19^+^ B cells. **(B)** Comparison of the percentages of IgG^+^ B cells between R (n = 17) and NonR (n = 33) NSCLC patients receiving anti-PD-1 monotherapy at the baseline. **(C)** The Kaplan-Meier analysis of the associations of the percentages of IgG^+^ B cells with immunotherapy PFS. The Wilcoxon test was used to analyze the differences between two groups. Survival curves were plotted by using the Kaplan-Meier method using median as the cutoff to define the high and low group. *P*-values were calculated by the log-rank statistics in Kaplan-Meier analyses. *: *P* < 0.05.


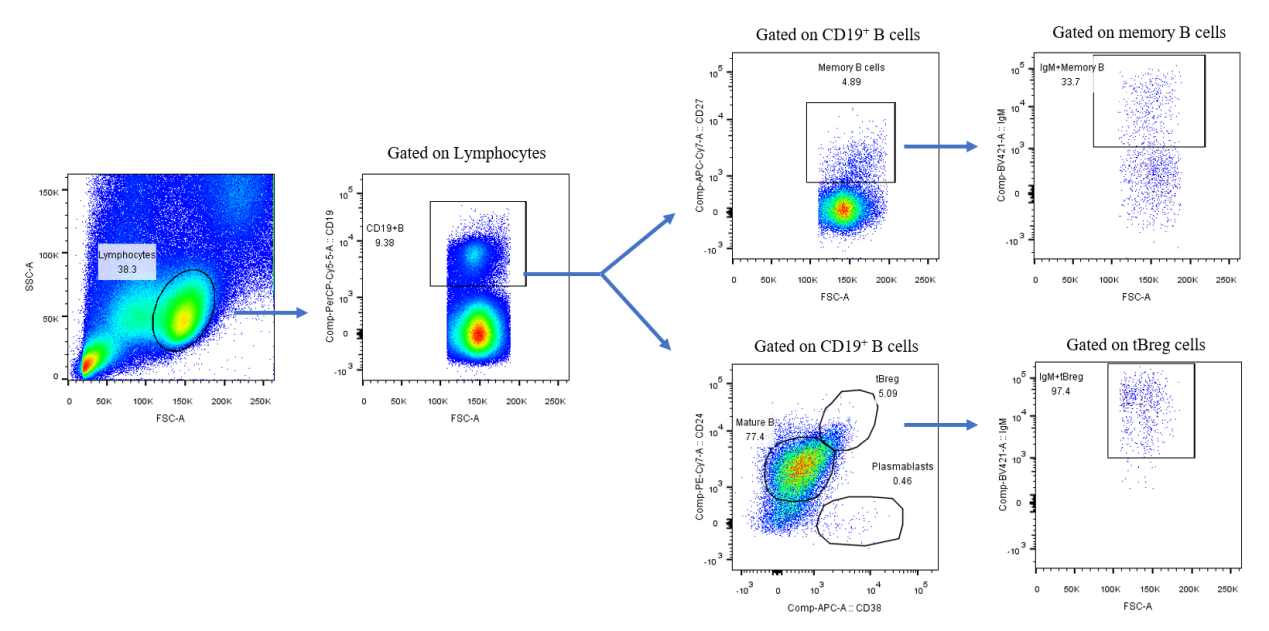


**FIGURE S****2** The gating strategy of IgM positive cells in B cell subsets. Memory B cells (CD27^+^CD19^+^ B), mature B cells (CD24^+^CD38^-^CD19^+^ B), transitional regulatory B cells (tBreg, CD24^++^CD38^+^CD19^+^ B) and plasmablasts (CD24^-^CD38^+^CD19^+^ B) were gated on CD19^+^ B cells.


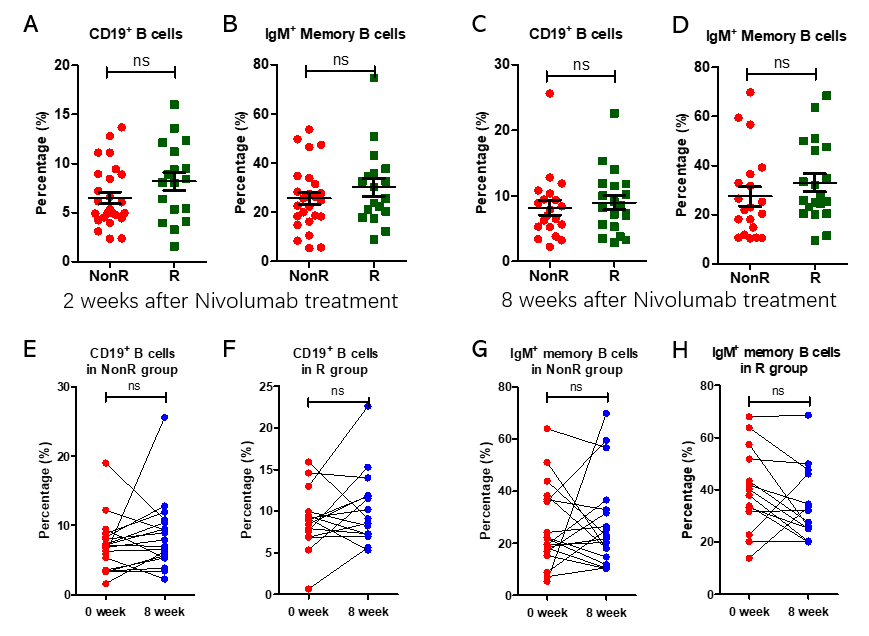


**FIGURE S3** Dynamics of peripheral CD19^+^ B and IgM^+^ memory B cells after anti-PD-1 monotherapy. **(A-D)** Comparison of CD19^+^ B and IgM^+^ memory B cell percentages between R and NonR patients at 2 weeks **(A, B)** and 8 weeks **(C, D)** after anti-PD-1 monotherapy. **(E-H)** The percentages of CD19^+^ B **(E, F)** and IgM^+^ memory B cells **(G, H)** at the baseline and 8 weeks after nivolumab treatment in NonR (n = 18) and R (n = 14) patients. The paired-*Student t* test was used to analyze the differences in baseline and after the treatment. ns: *P* > 0.05.


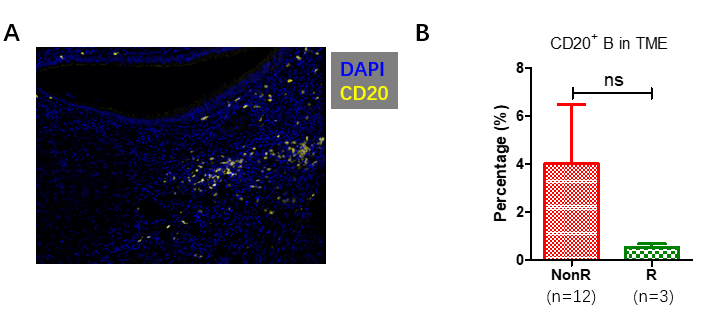


**FIGURE S4** Tumor-infiltrating B cells were tested by multiplex immunofluorescence. **(A)** The image of CD20^+^ B cells in tumor measured by multiplex immunofluorescence. **(B)** Comparison of Tumor-infiltrating B cell percentages between R (n = 3) and NonR (n = 12) patients receiving anti-PD-1 monotherapy. ns: *P* > 0.05.


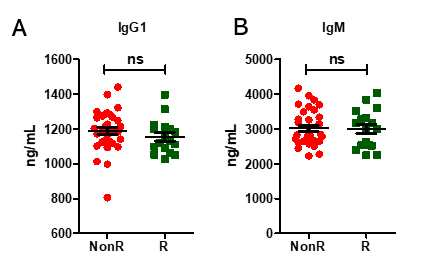


**FIGURE S5** Comparison of plasmatic IgG1 **(A)** and IgM **(B)** between R (n = 17) and NonR (n = 33) NSCLC patients at the baseline. ns: *P* > 0.05.


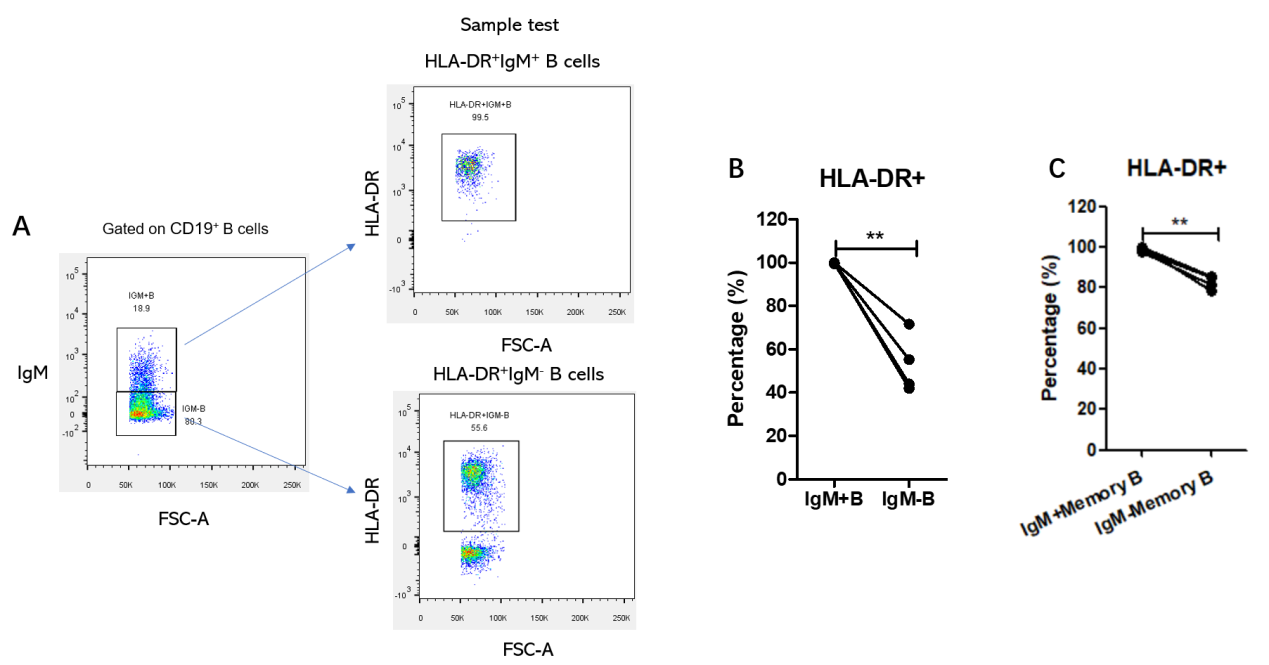


**FIGURE S6** IgM^+^ B cells demonstrate high expressions of HLA-DR. **(A)** The gating strategy of MHC class II molecules HLA-DR positive cells in IgM^+^ B and IgM^-^ B cell subsets. **(B)** Comparison of HLA-DR expressions between IgM^+^ B and IgM^-^ B cells in 4 advanced NSCLC patients receiving anti-PD-1 monotherapy at the baseline. **(C)** Comparison of HLA-DR expressions between IgM^+^ memory B and IgM^-^ memory B cells. **: *P* < 0.01.


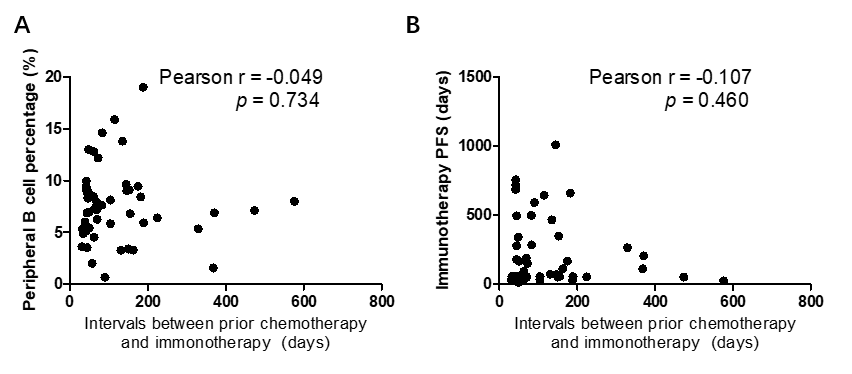


**FIGURE S7** Association analysis of the interval between prior chemotherapy and immunotherapy with baseline B cell percentages **(A)** and immunotherapy PFS **(B)**.


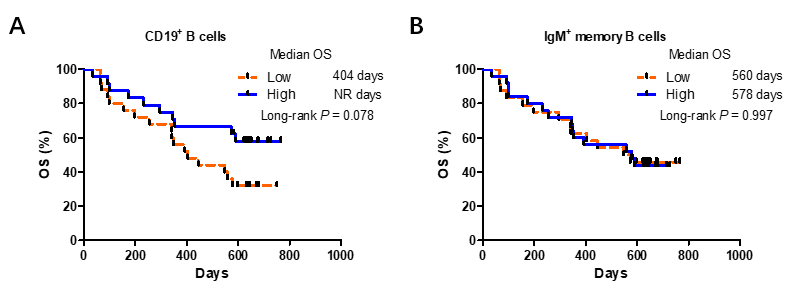


**FIGURE S8** The Kaplan-Meier analysis of the associations of percentages of CD19^+^ B **(A)**, IgM^+^ memory B **(B)** cells with anti-PD-1 monotherapy OS.

**TABLE S1** Antibodies used in multi-color flow cytometry

| **Targets** | **Fluorochrome** | **Source** | **Product code** |
| --- | --- | --- | --- |
| Panel A |  |  |  |
| Human CD3 | BV510 | BD Pharmingen, San Diego, CA, USA | 564713 |
| Human CD8 | PE-CY7 | BD Pharmingen, San Diego, CA, USA | 557746 |
| Human CD19 | BV605 | BD Pharmingen, San Diego, CA, USA | 562653 |
| Human CD16 | Percp-cy5.5 | BD Pharmingen, San Diego, CA, USA | 560717 |
| Human CD27 | APC-H7 | BD Pharmingen, San Diego, CA, USA | 560222 |
| Human PD-1 | PE | BD Pharmingen, San Diego, CA, USA | 557946 |
| Human PD-L1 | BV421 | BD Pharmingen, San Diego, CA, USA | 563738 |
| Panel B |  |  |  |
| Human CD19 | Percp-cy5.5 | BD Pharmingen, San Diego, CA, USA | 561295 |
| Human CD27 | APC-H7 | BD Pharmingen, San Diego, CA, USA | 560222 |
| Human CD38 | APC | BD Pharmingen, San Diego, CA, USA | 555462 |
| Human CD24 | PE-CY7 | BD Pharmingen, San Diego, CA, USA | 561646 |
| Human PD-1 | BV650 | BD Pharmingen, San Diego, CA, USA | 564104 |
| Human PD-L1 | PE | BD Pharmingen, San Diego, CA, USA | 557924 |
| Human IgM | BV421 | BD Pharmingen, San Diego, CA, USA | 562618 |
| Human IgG | PE-CF594 | BD Pharmingen, San Diego, CA, USA | 563246 |

**TABLE S2** Clinical characters between R and NonR patients in cohort 1

| **Characteristics** | **NonR (n = 33)** | **R (n = 17)** | ***P*** |
| --- | --- | --- | --- |
| Age, y  Median | 63 | 60 | 0.176 |
| Sex, n (%)  Male  Female | 26 (78.8)  7 (21.2) | 15 (88.2)  2 (11.8) | 0.663 |
| History, n (%)  Squamous  Non-squamous | 12 (36.4)  21 (63.6) | 5 (29.4)  12 (70.6) | 0.623 |
| Smoking status, n (%)  Smoker  Nonsmoker | 25 (75.8)  8 (24.2) | 14 (82.4)  3 (17.6) | 0.863 |
| Disease stage, n (%)  III  IV | 3 (9.1)  30 (90.9) | 5 (29.4)  12 (70.6) | 0.147 |
| EGFR mutation, n (%)  Yes  No  UN | 5 (15.2)  23 (69.6)  5 (15.2) | 1 (5.9)  13 (76.5)  3 (17.6) | 0.632 |
| Treatment, n (%)  Second-line  More than second | 29 (87.9)  4 (12.1) | 16 (94.1)  1 (5.9) | 0.842 |

**TABLE S3** Univariate and multivariate logistic regression analyses of B cell and CD4^+^ T cell signatures in the prediction of the responses to anti-PD-1 monotherapy

| **Factors** | **Univariate**  **(Kaplan–Meier test)** | | **Multivariate**  **(Logistic regression)** | |
| --- | --- | --- | --- | --- |
|  | ***P* value** | **HR (95% CI)** | ***P* value** | **HR (95% CI)** |
| CD19^+^ B cells | 0.002 | 2.97 (1.52-5.84) | 0.373 |  |
| IgM^+^ B cells | 0.004 | 2.63 (1.36-5.10) | 0.614 |  |
| IgM^+^ Mature B cells | 0.012 | 2.33 (1.21-4.50) | 0.963 |  |
| IgM^+^ Memory B cells | 0.003 | 2.72 (1.39-5.30) | 0.002 | 1.09 (1.03-1.16) |
| IgM^+^ tBreg cells | 0.004 | 2.68 (1.38-5.20) | 0.379 |  |
| IgM^+^ Plasmablasts | 0.008 | 2.44 (1.27-4.70) | 0.628 |  |
| CD45RO^+^CD4^+^ T | 0.028 | 2.11 (1.11-4.02) | 0.018 | 1.06 (1.01-1.12) |
| CD45RO^+^CTLA-4^+^CD4^+^ T | 0.010 | 2.36 (1.23-4.53) | 0.750 |  |
| CD45RO^+^PD-1^+^CD4^+^ T | 0.045 | 1.73 (0.92-3.26) | 0.648 |  |
| CD45RO^+^PD-L1^+^CD4^+^ T | 0.019 | 2.43 (1.16-5.10) | 0.380 |  |
